# Accelerating diagnosis of degenerative cervical myelopathy through improved education: a mixed-methods study protocol from Myelopathy.org RECODE-DCM to define stakeholders, knowledge requirements and an optimal intervention strategy

**DOI:** 10.1136/bmjopen-2025-107940

**Published:** 2026-03-24

**Authors:** Munashe Veremu, Naomi Deakin, Rohil V Chauhan, Justin M Lantz, Georgios Toumbas, Julia Tabrah, Vishal Kumar, Carl M Zipser, Joshua Plener, Carlo Ammendolia, David B Anderson, Ellianne J dos Santos Rubio, Lindsay Tetreault, Rahul Parnaik, Ricardo Rodrigues-Pinto, Otieno Martin Ong’wen, Ellen Sarewitz, Iwan Sadler, Theresa Roberts, Neil Langridge, Gabrielle Swait, Lisa Hadfield-Law, Jerry Draper-Rodi, Lianne Wood, Sybil Stacpoole, Mario Ganau, Shehla Baig, Antony Bateman, Andreas K Demetriades, Willem Cornelis Wilco Peul, Benjamin Davies

**Affiliations:** 1 Department of Clinical Neurosciences, University of Cambridge, Cambridge, UK; 2 Active Living and Rehabilitation: Aotearoa New Zealand, Health and Rehabilitation Research Institute, Faculty of Health and Environmental Sciences, Auckland University of Technology, Auckland, New Zealand; 3 Auckland Spine Surgery Centre, Auckland, New Zealand; 4 Division of Biokinesiology and Physical Therapy, Keck School of Medicine, University of Southern California, Los Angeles, California, USA; 5 Myelopathy.org, Cambridge, UK; 6 Hounslow and Richmond Community Healthcare NHS Trust, Teddington, UK; 7 Department of Orthopaedics, PGIMER, Chandigarh, India; 8 Spinal Cord Injury Center, Balgrist University Hospital, University of Zurich, Zürich, Switzerland; 9 Department of Anesthesiology and Pain Management, Mount Sinai Hospital, Toronto, Ontario, Canada; 10 Institute for Health Policy, Management and Evaluation, University of Toronto, Toronto, Ontario, Canada; 11 School of health sciences, The University of Sydney, Sydney, New South Wales, Australia; 12 Sydney Musculoskeletal Health, Faculty of Medicine and Health, The University of Sydney, Sydney, New South Wales, Australia; 13 Department of Neurosurgery, Curaçao Medical Center, Willemstad, Curaçao; 14 Department of Surgery, University of Toronto, Toronto, Ontario, Canada; 15 Department of Medicine, University College Cork, Cork, Ireland; 16 St Mary’s Surgery, NHS, Ely, UK; 17 Spinal Unit (UVM), Department of Orthopaedics, Centro Hospitalar Universitário do Porto EPE, Porto, Portugal; 18 School of Medicine and Biomedical Sciences, University of Porto, Porto, Portugal; 19 Hospital CUF Trindade, Porto, Portugal; 20 Kenya Association of Physiotherapists, Nairobi, Kenya; 21 Nursing education, Elsevier Inc, New York, New York, USA; 22 Health Sciences University, Bournemouth, UK; 23 Royal College of Chiropractors, Reading, UK; 24 Coventry University, Coventry, England, UK; 25 Independent Surgical Educationalist, Charlbury, UK; 26 Health Sciences University—London Campus, London, UK; 27 University of Exeter, Exeter, UK; 28 Neurology Unit, Department of Clinical Neurosciences, University of Cambridge, Cambridge, UK; 29 School of Medicine, BAU International University Batumi, Batumi, Georgia; 30 Nuffield Department of Clinical Neurosciences, University of Oxford, Oxford, UK; 31 City St George’s University of London, London, UK; 32 Royal Derby Spine Centre, Royal Derby Hospital, Derby, UK; 33 Edinburgh Spinal Surgery Outcomes Study Group, Department of Neurosurgery, University of Edinburgh Western General Hospital, Edinburgh, UK; 34 Neurosurgery, Leiden University Medical Center, Leiden, The Netherlands

**Keywords:** NEUROSURGERY, NEUROLOGY, MEDICAL EDUCATION & TRAINING, Health Education

## Abstract

**Abstract:**

**Introduction:**

Outcomes for degenerative cervical myelopathy (DCM) patients are limited by delayed and missed diagnoses, driven in part by poor professional awareness. Despite DCM being the most common cause of adult spinal cord injury, it remains under-recognised and undertaught in clinical education. Lessons from other common pathology like stroke and acute myocardial infarction highlight the potential of education to improve early diagnosis. This study will develop a professional education strategy to improve early DCM diagnosis. It will define key audiences and identify an effective delivery method, laying the groundwork for a sustained, targeted intervention.

**Methods and analysis:**

The study aims to define who needs to know about DCM, what they need to know and how they can learn it. This will be carried out in three phases: phase 1—who and what: to establish the target population and to define core competencies for the educational intervention; phase 2—how: to create and review the educational intervention; phase 3—evaluation: to test whether the framework is an improvement to existing strategies.

**Ethics and dissemination:**

Ethical approval is in place from the University of Cambridge (HBREC.2024.24). Results from the study will be disseminated through scientific publication, conference presentation, blog posts and podcasts.

**PROSPERO registration number:**

CRD42023461838

**STRENGTHS AND LIMITATIONS OF THIS STUDY**

- Novel methodology for designing an educational intervention for degenerative cervical myelopathy (DCM), and the framework described could be used for other diseases with a need for professional education.
- Mixed-methods, multiphase design incorporating interviews, Delphi consensus, systematic review and stakeholder workshops enable comprehensive and theory-informed development of the educational intervention.
- Strong international, multidisciplinary and patient coproduction through the steering group and consensus meetings enhances relevance to real-world DCM diagnostic pathways.
- Use of predefined consensus methodology, intervention mapping and transparent analytic approaches improves methodological rigour and reproducibility.
- Restriction to English-speaking participants and focus on high-income healthcare settings may limit generalisability to lower resource or non-English-speaking contexts.

## Introduction

 Degenerative cervical myelopathy (DCM) is a preventable cause of long-term disability. Due to chronic cervical cord compression (either from degenerative, traumatic or congenital causes), patients present with neck or arm pain, sensorimotor impairment and/or autonomic disturbances, which can progress to paralysis.^1 2^ With an estimated adult prevalence of 2%,^3^ DCM is the most common cause of adult spinal cord injury worldwide; however, its diagnosis is often delayed. A recent observational study revealed that nearly 80% of patients with DCM were diagnosed late (>6 months).^2^ However, the real magnitude of the problem is likely even larger, since such studies overlooked individuals without a diagnosis. Specifically, a recent UK study estimated that less than 10% of incident DCM is formally diagnosed.^4^ Due to the progressive nature of the disease, and because surgery is the most effective treatment despite recent advancements,^5 6^ both delayed and missed diagnoses can lead to irreversible disability and subsequently poor outcomes for people living with DCM (pwDCM).^7^ Poor awareness of DCM, which has been identified internationally across professional clinical groups,^8^,^10^ is thought to perpetuate the delays in the diagnosis of DCM.^11 12^ DCM disproportionately affects the working age population and is a cause of early retirement due to disability. In older populations, progressive neurological impairments from DCM increase support needs. Together, this translates into significant socioeconomic costs, estimated at approximately £700 million per year due to lost earnings and need for social support.^13^ Thus, efforts to improve early diagnosis may help prevent avoidable and significant long-term disability.

Increasing awareness through professional education to providers and patients can help to reduce the burden of disease and improve patient outcomes.^12^ For example, breast cancer care, which has established professional and public health awareness programmes, has recently been shown in a multicentre prospective cohort study to reduce mortality rates across a 20-year period (1993–2015).^14^ While advances in treatment technology and breast cancer research are undeniably significant contributors, educating professionals about ‘red-flag’ symptoms as well as increased breast cancer awareness through prescribed national screening programmes likely to have contributed to the described success.^15^

Another group of patients who have benefited from increased awareness are acute myocardial infarction (AMI) and stroke patients, who were previously treated with supportive therapy in the 20th century, which resulted in high morbidity and mortality rates.^16 17^ Through advancements in investigation (such as vascular imaging and measurement of cardiac enzymes), treatment (including primary/secondary prevention and revascularisation therapy)^18^ and education (lay titles such as ‘heart attack’ and well-known typical symptoms like the FAST (Face, Arms, Speech, Time) acronym), survival outcomes have improved.^19^ Breast cancer, stroke and AMI are all diseases which, similar to DCM, may initially present to a non-specialist practitioner. Education provided to non-specialists, to better recognise and appropriately triage patients with potential breast cancer, stroke or AMI has likely contributed to improved outcomes for these patients; the same intervention and outcome is undoubtedly achievable for patients with suspected DCM.

Missed diagnosis and diagnostic delay are thought in part to be driven by a lack of professional knowledge and awareness of DCM. In some settings, patient factors (beliefs around the role of surgery and trust in the healthcare system) and resource limitations may also contribute to this diagnostic delay. Patients on average will require five clinic consultations before a diagnosis is made, with average delay to diagnosis of 2.5 years.^20 21^ From the patient’s perspective, patients with a diagnosis of DCM report that non-specialists frequently have ‘never heard of the condition’.^22^ In the sphere of healthcare education, previous studies have shown that DCM is infrequently featured in educational curricula and learning resources for various healthcare professions,^9 23^ and that knowledge is incomplete even among medical students.^8^ Furthermore, of the continuing professional development (CPD) resources that exist, most published DCM education focuses on the management of the condition, rather than diagnosis.^24^ Given that diagnostic delay has been identified as main contributors to a poor outcome in DCM,^21^ it is essential to educate healthcare professionals involved within its diagnostic pathway. Additionally, improving DCM awareness was identified as the number one research priority by AO Spine Research Objectives and Common Data Elements for Degenerative Cervical Myelopathy (RECODE-DCM).^19^ The AO Spine RECODE-DCM community represents an international group of clinicians and researchers dedicated to improving the care of DCM patients. This study aims to develop an educational intervention to improve professional awareness of DCM. To achieve this, it must first identify the target population and the most effective approach to reach them. We will review educational interventions for neurological disease in order to identify approaches that can be included in our intervention. DCM patients present with neurological symptoms, and the previously documented problem of ‘neurophobia’ (the fear and difficulty in understanding neurological diseases) among students further stresses the importance of exploring approaches to educational interventions within other neurological diseases.^25^

Through process mapping, we identified that a diverse range of professionals are involved in the diagnosis of DCM, including primary care practitioners (eg, general practitioners, physiotherapists), secondary care practitioners (neurologists, rheumatologists, pain specialists and geriatricians), other musculoskeletal (MSK) clinicians (including, but not limited to, chiropractors, osteopaths, hand therapists, nurse practitioners, MSK medicine physicians, sports medicine physicians, urgent care doctors, general orthopaedic surgeons physical medicine and rehabilitation physicians) and orthopaedic or neurosurgical spine surgeons.^20^ While education has been identified as a key intervention to improve DCM outcomes, early initiatives, including those by Myelopathy.org, indicated that ‘what’ stakeholders need to know with respect to their role in diagnosis, and ‘how’ they wish to learn or be educated varies considerably. We recently conducted a small-scale educational pilot project involving physiotherapists and neurosurgeons collaborating to design a PowerPoint training package for General Practice (GPs) and Musculoskeletal (MSK) physiotherapists. The package encompassed an overview of pathology, common symptoms, clinical tests, triage principles and treatment guidelines, supplemented by interactive case studies for triage practice. Delivery occurred virtually through lunchtime and evening workshops for clinicians in West London. Pretraining and post-training self-assessment surveys were administered to measure DCM awareness and triage confidence and revealed a significant increase across all GP and physiotherapy participants, with positive feedback. While effective for a limited group, virtual training remains time-consuming, labour-intensive and lacks universal applicability.^26^ A recent UK national cross-sectional study led by our team on GP awareness of DCM showed GPs lack confidence in the recognition and management of DCM.^27^ Further work is therefore needed to understand how educational resources can match the learning needs of different professional groups with varying levels of experience in an efficient and effective way. This knowledge gap is a significant hurdle to raising DCM awareness, for a relatively resource-poor condition where efficiency is paramount.^19 28^

To address this unmet educational need, a working group has been formed to develop a grounded educational strategy for diagnosis in DCM, utilising Myelopathy.org and its RECODE-DCM community. First, we will define the target audience for the educational initiative and outline their learning needs. second, we will describe how these groups could be reached and how intervention efficacy will be measured.

In this way, this project will lay strong foundations for a sustained, targeted and efficient educational intervention in DCM.

## Methods

### Overview and scope

This project aims to define the learning needs and mode of education for the professional groups involved in the diagnosis of DCM. Recognising differences in healthcare delivery, this first initiative will focus on being representative of, and applicable to, high-income countries (HIC), particularly those with consistency in workforce and infrastructure with necessary surgical interventions readily available. This project will be delivered by a steering group (detailed in table 1).

**Table 1:** Glossary

**Term**	**Description**
Consensus meetings	Consensus meetings will be conducted during particular phases of the study, such as to discuss a project aim, and will include external stakeholders in addition to members of the steering group.
CPD	An acronym for continuing professional development.
Diagnostic roles	The role that a stakeholder or healthcare professional has for diagnosing DCM.
Educational intervention	Introducing a new teaching method to improve understanding of DCM in order to improve its diagnosis.
Educational strategy	A structured plan that defines the target learners, learning objectives and delivery approaches needed to improve professional understanding and diagnosis of DCM.
Educational framework	An organised structure that guides the development, implementation and evaluation of the educational intervention to improve diagnosis of DCM.
Management group	A group within the steering group that will carry out the day-to-day management of the project, which includes planning for concurrent and future phases of the study, data analysis and writing manuscripts for dissemination.
Non-specialist	A healthcare professional who sees undifferentiated patients at first contact, without specific expertise in spinal conditions. This includes generalists such as GPs, emergency clinicians and nurse practitioners. In this context, non-specialists are distinct from clinicians with specialist training in spinal conditions, such as specialist spine physiotherapists.
Persona	With respect to diagnosing DCM, a professional group who needs to know the same information and uses the same educational tools.
RECODE-DCM community	An acronym for REsearch objectives and COmmon Data Elements for Degenerative Cervical Myelopathy; an international community of professionals and researchers dedicated to improving the care of DCM patients hosted by Myelopathy.org.
Steering group	A group comprising professionals, individuals with DCM and study investigators who will convene quarterly, with additional meetings held at the project’s inception and conclusion. The steering group will also be invited to consensus meetings.
Study investigator	A healthcare professional/researcher who is leading a particular phase of the study.

CPD, continuing professional development; DCM, degenerative cervical myelopathy.

We will use the results of the early stages of this study to design preliminary education interventions that satisfy the educational needs of healthcare professionals working within the DCM diagnostic pathway. This is principally to demonstrate how the framework can be used to design an intervention.

**Figure 1:**
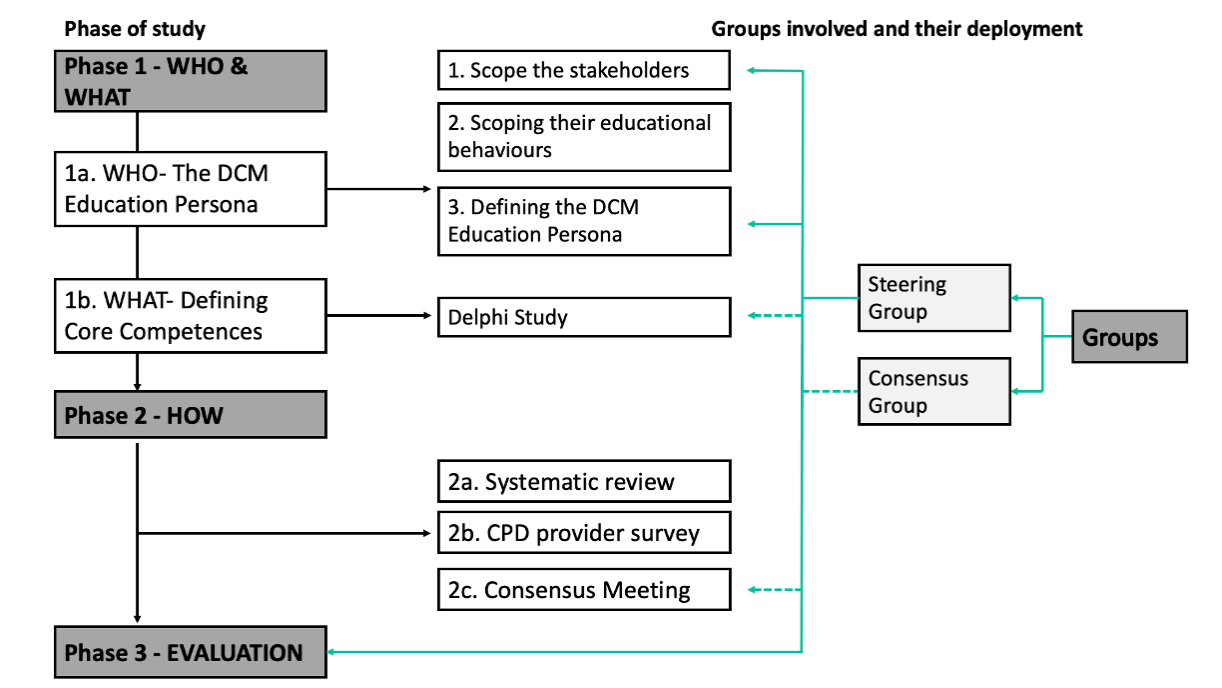
Overview of phases for the development of an educational intervention for DCM. Phase 1 answers who the target population is and what should be taught, phase 2 answers how the educational intervention will be delivered and phase 3 evaluates the findings from phases 1 and 2 into an educational intervention. On the right, there are the different groups with green arrows indicating their inclusion within a study. Checked arrows represent the involvement of the consensus group. Non-checked arrows represent the steering group involvement. CPD, continuing professional education; DCM, degenerative cervical myelopathy.

This project will be conducted in three phases (summarised in figure 1 and table 2):

- **Phase 1—Who and what:** to establish the target population and to define core competencies for the educational intervention.
- **Phase 2—How:** to create and review the educational intervention.
- **Phase 3—Evaluation:** to test whether the framework is an improvement to existing strategies.

**Table 2:** Status of project as of February 2026

Phase	Status	Description and estimated time of completion (ETC)
1	In progress	Phase 1a and 1b in progress. ETC October 2026.
2	In progress	2a (systematic review of literature) completed in June 2025 and published in November 2025. 2b and 2c in planning. ETC March 2027.
3	In planning	Strategy to be refined with results from phases 1 and 2.

The start date of the project: January 2025. The anticipated completion date will be in July 2027.

### Patient and public involvement

This study will be coproduced with pwDCM throughout; at least two pwDCM will be represented within the steering group.

### Project oversight and management

A global steering group will lead the DCM educational initiative. The group will consist of national (UK) and international academic spinal surgeons, academic doctors, GPs and allied healthcare professionals (AHPs; such as physiotherapists, chiropractors, osteopaths and advanced care practitioners), educational researchers and at least two patient representatives. The ideal ratio is 1:1 representation between surgeons and other healthcare professionals, with at least more than one geographical region (Latin America, North America, Middle East and Africa, Europe, Asia/Pacific) represented within each professional group. The group will meet at the beginning and end of the project. In addition, quarterly meetings will be used to discuss project progress and supported by ad hoc consensus meetings. Decisions at steering group meetings must be made by majority voting. Furthermore, a meeting must be considered quorate, meaning at least one study investigator, one patient representative and two professionals involved in the diagnostic pathway for DCM (Surgeon and/or other Healthcare professional) are present. Day-to-day delivery of the project will be led by the management group.

### Study participants

#### Inclusion criteria

All participants will be English-speaking adults (aged ≥18 years old) with access to the internet.

#### Stipulated group membership

##### Management group

The management group will consist of four members who will carry out the day-to-day study activities and will include all principal and study investigators.

##### Steering group

Membership will be defined according to the following eligibility criteria: individuals who (1) have published articles in peer-reviewed journals on the topic of the diagnosis or management of DCM; (2) possess significant clinical experience (at least 10 years) in assessing, diagnosing and/or treating patients with DCM or similar neurological conditions; (3) regularly evaluate undifferentiated patients (patients without a formal diagnosis who are subsequently triaged to a specialty by a non-specialist practitioner); (4) have significant experience in delivering educational interventions; or (5) have personal experience living with the disease. There are no exclusion criteria. Eligibility will be assessed by the management group.

##### Consensus meetings

**Figure 2:**
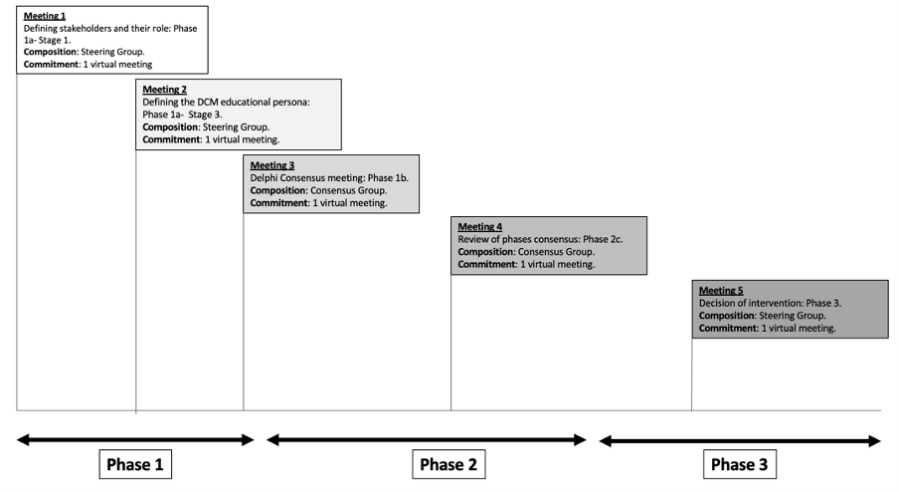
Timeline meeting schedule for the three phases of the project, with the composition and commitment for each meeting. DCM, degenerative cervical myelopathy.

While consensus decisions may be reached by the steering group, specific stand-alone consensus meetings will be planned for specific aspects of this project, such as the Delphi survey (phase 1b) and finalising the educational intervention (phase 2c). These are referred to as ‘consensus meetings’. All meetings and their respective composition are shown in figure 2. A consensus meeting must have at least 40% representation from stakeholders who are external to any of the project committees. These stakeholders will be identified from the RECODE-DCM community, a community of healthcare professionals and individuals with lived experience of the disease, dedicated to improving care for DCM patients.

Consensus meetings will aim to have representation from surgeons, neurologists, musculoskeletal practitioners and patients from at least three continents. Other stakeholder groups are eligible to participate if there is availability and if they are deemed relevant by the steering group. Overall, surgeons should make up less than 40% of the group membership.

### Ethics

The University of Cambridge Human Biology Research Ethics Committee has approved the study (HBREC.2024.24). All data will be handled according to University of Cambridge Data Handling Policy.^29^

### Recruitment

The participants will be recruited by email from the RECODE-DCM community.

### Consent

All participants will complete electronic consent (consensus/workshop/interview/survey) and will be asked to acknowledge any relevant conflicts of interest (COI) during each phase. Electronic consent will be a two-step process. Participants will be asked to read and electronically sign a participant information sheet (PIS) as well as declare any COI via a click-to-confirm button, which will take them to a short registration survey. The PIS will include all necessary information for the study and depending on the phase of the study and will ask for permission to record semistructured interviews for analysis. After clicking to confirm, participants will be submitting their consent to participate in that particular study. Members of the steering group have already consented to project involvement.

### Contributorship/authorship and dissemination

Participation will be incentivised through acknowledgement and authorship; specifically, any stakeholder who contributes will be acknowledged in relevant publications, with authorship reserved for steering group members who attend at least 50% of meetings, or stakeholders who participate in at least two consensus meetings (or in one meeting that forms a significant step within a phase of the study). Results from the study will be disseminated through scientific publication, conference presentation, blog posts and podcasts on the myelopathy.org website.

### Phases

#### Phase 1a: who should the target population be, and what are their goals?

Aim: to define the DCM professional education ‘persona’

Deliverable: a set of mutually exclusive groups, built around their ‘role’ (ie, what they must instigate in order to facilitate a diagnosis) in the diagnosis of DCM.

A ‘persona’ is a term frequently utilised commercially, where products are designed and optimised to a population who possess certain traits and characteristics. Phase 1a will be conducted in three stages with the ultimate aim of defining the DCM professional education ‘persona’:

1. Stage 1: scope the stakeholders; prioritise and define their roles.
2. Stage 2: scope stakeholder educational preferences.
3. Stage 3: define the DCM educational persona.

##### Stage 1: scope the stakeholders; prioritise and define their roles

Using electronic health records, a UK study investigated the diagnostic pathway of DCM patients and identified a convoluted pathway with multiple stakeholders.^20^ Using this as a framework, a process mapping exercise informed by both patient and professional interviews is currently underway.^30^ Specifically, patients will be interviewed on the key decision-making processes within their DCM diagnosis journey, capturing the professionals involved within this journey. Data collection follows a spiral process where information is collected until the point of information saturation—where no new information arises from the interviews.^31^ We will use the results of this process mapping exercise, alongside feedback from the steering group, to define a longlist of professionals involved in the diagnosis of DCM. This will follow a nominal group technique (NGT), with members first submitting suggestions independently via a premeeting online survey, followed by formal discussion at a steering group meeting. The list of professionals will either be approved, or the process will be repeated until a majority is reached. The steering group will also prioritise this list, as pragmatically it may not be feasible to scope across an exhaustive list of all the stakeholders a patient may encounter.

With reference to the process map and list of stakeholders, the steering group will develop a mutually exclusive list of draft roles within DCM diagnosis. The subsequent education initiative will focus on action within the diagnostic pathway based on these predefined stakeholder roles. For example, the role of a GP may be to suspect a diagnosis of DCM and to make a referral to secondary care. This process will similarly follow an NGT, with members submitting suggestions, which will be initially reconciled by the management group, before presenting to the steering group. As outlined above, the list of roles will either be approved, or the process repeated until a majority is reached. Finally, the roles will be further externally validated in Stage 2.

###### Role of students

Students will be included in this study. Medical students will be a group of their own, and AHP students will be encompassed within each profession.

### Stage 2: scoping their educational behaviours

Prioritised stakeholders identified in stage 1 will be approached, using purposeful sampling, for 1:1 interviews and/or group workshops to:

1. Validate the defined role (identified in stage 1).
2. Establish required information to achieve their goal within DCM diagnosis.
3. Establish their educational behaviours and current methods of CPD.

#### Recruitment and sampling

The participant recruitment and consent procedure is detailed in the relevant sections above. We will aim to recruit a 1:1 ratio of the prioritised stakeholders identified in stage 1. We will include international participants (South America, North America, Middle East and Africa, Europe, Asia/Pacific). Similarly, the stop criteria will be information saturation.^31^ After registration and consent, participants will be invited to a virtual 1:1 interview and/or group workshop.

#### Question development

Participants will be asked a set of predefined questions by a study investigator. Survey questions (the ‘topic guide’) will be iteratively developed by the management group prior to a review in a meeting with the steering group. Questions will be divided into three sections that: (1) validate the previously defined role for that professional; (2) determine information needed to perform their role and (3) ascertain their methods of CPD. Given that the questions will be aimed at a wide range of healthcare professionals, the topic guide will be designed as a series of open followed by hierarchical questions (where one question leads to the next, chronologically from sections 1–3). Each open question will have a list of generic prompts that could be applicable to any healthcare professional. Using section 3 (preferred method of CPD) as an example, the first question would be open and use prompts, including (but, not limited to) podcasts, in-person workshops and massive open online courses. The follow-up hierarchical question would similarly be open and would explore the reasons for choosing that CPD method. Subsequent follow-up questions would elucidate who currently provides their CPD and will also canvass positive and negative experiences with CPD. Questions within sections i and ii will follow the described open and hierarchical format.

#### Procedure, data analysis and output

A study investigator will perform the semistructured interviews using a virtual videoconferencing application (such as Zoom/Microsoft Teams/Google Meet). Interviews will be recorded and transcribed verbatim using previously described methodology.^32^ Data collection will continue until information saturation, which will define the stop criteria for recruitment. Audio transcripts will be analysed using inductive thematic analysis to generate themes as previously described by two independent coders. Conflicts will be resolved with discussion, and discussion with senior author (BD) if a consensus is not reached.^32 33^ Frequency statistics will be used to explore theme popularity, potentially allowing for comparisons between groups of professionals, using either parametric or non-parametric tests depending on the derived dataset.^34^ The ambition is to produce one journal article paper, per stakeholder group, detailing the knowledge requirements and CPD behaviours, including a subgroup analysis of variation by region (South America, North America, Middle East and Africa, Europe, Asia/Pacific), and by stage of career (student/trainee vs fully qualified).

### Stage 3: defining the DCM educational persona

A persona is a term frequently utilised commercially and refers to designing products tailored to a target population’s specific traits and characteristics. In applying the persona concept to our intervention, we aim to identify the key and homogenous education profile(s) within the diagnostic pathway for DCM. Ultimately, the aim is to not only improve effectiveness, by identifying more homogenous groups, but also efficiency, as it is anticipated some persona will cross disciplines, enabling interventions to have broader relevance.

To achieve this, we will need to understand the key traits (including knowledge requirement and their role within DCM diagnosis) of professionals within the DCM pathway. Through this exercise, we will group different types of professionals within a single persona based on their common traits, which may reveal a common educational need. With this common persona, we can then share education resources and strategies broadly between professionals.

The development of a persona will be achieved using a consensus meeting workshop with the members of the steering group. Prior to the meeting, the management group will generate a list of personas based on the results of stage 2, where commonalities in: (1) knowledge requirement; (2) the role in diagnosing DCM and (3) the ability to access diagnostic investigations will be used to group professionals into the key persona requiring education for DCM diagnosis.

#### Consensus meeting procedure

The meeting will be convened as a moderated virtual group task following an NGT (as previously described), where the generated personas will be voted on by the meeting attendees. Attendees will be asked to vote on each persona using a scale from 1 to 9 (1=inappropriate, 9=appropriate). The pre-established consensus criteria for determining the inclusion of a proposed persona will be as follows: if all ratings fall within the range of 1 to 3, it will be deemed unanimously inappropriate for inclusion; if ratings range from 4 to 6, it will be considered equivocal for inclusion; and if all ratings range from 7 to 9, it will be collectively agreed on as appropriate for inclusion as a suggested persona.^35^ The meeting will be run using a videoconferencing application, with breakout rooms for initial discussions and subsequent voting. Results from each breakout room will be shared in a plenary session, where disagreements between rooms can be discussed in order to reach consensus. If consensus is not reached, further moderated discussion will take place and revoting will be repeated until consensus is achieved. Final results of the persona forming exercise will be disseminated through email to the attendees of the consensus meeting.

### Phase 1b: what are the knowledge requirements to achieve these goals? Defining core competencies for DCM education.

Aim: What is the knowledge required to achieve these goals?

Deliverable: minimum knowledge requirements to achieve each goal.

Phase 1b will generate core competencies for the previously identified persona(s), which the educational intervention will need to satisfy. Phase 1b will be completed in two stages, with each occurring interchangeably or concurrently:

Generate a list of descriptors to inform the Delphi questionnaire:

The list of descriptors will be generated from a recent scoping review of educational resources in DCM.^24^ The information items will be categorised into a set of domains through consensus discussion with the steering group and will populate the Delphi questionnaire, which will be disseminated to a group of professionals involved in the diagnosis of DCM to generate a consensus for the core competences.

- Perform a modified Delphi study:

Given that the initial list of descriptors will be generated from previously established work, this will be a modified Delphi^36^; similar to the classic Delphi study but without the initial open-ended first round survey to generate the list of descriptors.

The primary outcome of this stage will be to define a set of core competencies for DCM diagnosis through consensus as well as the referral to definitive care. This set of core competencies will be used to direct the content of our educational initiative in phase 2c/3 and to inform future DCM educational initiatives. We will include intervention mapping (IM) exercises for the competencies designed.^37^ IM offers a structured approach to developing complex health interventions by integrating stakeholder expertise, best evidence and theory. In the context of DCM diagnosis, IM can guide the creation of tailored educational strategies for different healthcare professional groups by identifying specific learning objectives and related behavioural determinants (eg, knowledge, skills, attitudes). This process helps ensure educational content is focused, evidence-based and designed to effectively change practice behaviour. For example, within the scope of phase 1b, an IM exercise will ask what the barriers and facilitators to achieve the competency identified will be? Another question may be what needs to be learnt/changed (behaviours/attitudes/beliefs, self-efficacy, reduce fear, etc) to overcome the barriers and facilitate change to achieve the competency identified?

#### Delphi process

*Recruitment*. Participants involved in the diagnosis of DCM will be recruited from the RECODE-DCM network, aiming for a 1:1 ratio between spinal surgeons and other professionals involved in diagnosis (such as medical physicians and AHPs). The consent process will follow the process described above.

*Survey development*. The survey will be disseminated using an online survey platform (such as the Joint Information Systems Committee Online Survey (JISC, Bristol, UK)),^38^ with each survey round open for 4 weeks and follow-up reminder emails sent at weekly intervals. Previously agreed descriptors will be ranked by participants on a 6-point Likert scale (where 1=strongly disagree to 6=strongly agree), with opportunity for further comments or addition of new descriptors in a free text box at the end of each domain.

*Round 1*. On completion, results will be analysed by the steering group, with particular attention to potential new descriptors suggested by participants in the qualitative responses. A round 1 report will be generated and disseminated to the participants, inviting any further comments.

*Round 2*. New descriptors, descriptors without consensus and descriptors with amendments from feedback will be recirculated in a second round to respondents who responded to the first round. If consensus is not reached virtually after two rounds, the results from both rounds will inform a final virtual consensus meeting to finalise the consensus.

*Data analysis*. Descriptor medians and IQRs will be used to analyse the data. A priori consensus for an important descriptor is a median ≥5 (respondents either agreed or strongly agreed with the descriptor) with an IQR ≤1.5 (minimal heterogeneity among respondent answers).^39^

### Phase 2: how should the educational intervention be provided?

Aim: to establish how the educational intervention should be provided.

Deliverable: guidance on how to effectively deliver education to each persona.

Phase 2 will be completed in three stages utilising the results of phase 1 to create a targeted educational intervention. We will combine this information with two further studies: (1) a systematic review of the literature exploring educational interventions in neurological disease; and (2) survey of postgraduate CPD providers. The final part of phase 2 will consolidate the results of phase 1 and 2, providing recommendations to help inform phase 3.

#### Phase 2a: systematic review of the literature: educational interventions in neurological

A systematic review will be used to comparatively analyse previous educational interventions in neurological disease relevant to healthcare workers and students. In this context, a neurological disease will be defined as any pathology, of any aetiology, that affects the brain, spine or central/peripheral nervous system either independently or simultaneously. An educational intervention will be defined as any teaching innovation or event that aims to transfer knowledge to a population. The population of interest will be adult healthcare workers or students (>18 years old) learning about neurological disease. Studies will be identified using four databases: MEDLINE, EMBASE, CENTRAL (Cochrane) and CINAHL plus (EBSCO).^40^,^43^ The search strategy (supplementary data, online supplemental figure 1) will be refined using an iterative process. Study deduplication and initial screening of titles and abstracts will be conducted using Rayyan (Rayyan Systems, Cambridge, Massachusetts). Studies will be excluded if (1) they include participants learning about non-neurological disease, (2) the population consists of non-healthcare practitioners or the general public or (3) the study does not include an evaluation of the educational intervention. The outcomes of interest will be the effectiveness of the educational interventions. Within this study, we will also identify and analyse a subgroup of studies that focus on educational interventions that try to improve diagnosis of a specific neurological disease. We will use the information from the systematic review to inform our intervention targeted at the professionals involved in diagnosis. The review has been prospectively registered with PROSPERO (ID: CRD42023461838).

#### Phase 2b: survey of postgraduate CPD providers

CPD refers to the activities professionals undertake to develop and improve their skills and knowledge. Furthermore, a CPD provider is an entity that provides post-graduate education. We will compile a list of CPD providers, provided by our prioritised list of stakeholders (obtained from Phase 1b). The outcome will be to identify the common educational formats which formal CPD providers use, and to identify the current trends in educational content. This will help inform our educational intervention and ensure that it is relevant to current educational practice.

##### Survey development and dissemination

The survey questions will be developed and piloted by the steering group, with a focus on common educational formats and current trends. They will consist of multiple-choice and free-text questions, which will be hierarchical. The virtual CPD surveys will be conducted using platforms such as Google Survey (Google LLC, California, USA) and SurveyMonkey (SurveyMonkey Inc, California, USA). A discussion within the steering group will define a prioritised list of CPD providers to be targeted, aiming for a 3:1 ratio of CPD provider per represented healthcare professional. The prioritised list of CPD providers will be approached using email and invited to complete a short survey.

##### Data analysis

Data will be analysed using descriptive and frequency statistics. Free text will be analysed using thematic analysis to generate themes and common concepts. The survey end will be defined as information saturation.^26^

### Phase 2c: final consensus meeting (summarising phases 1 and 2)

Using a virtual consensus meeting and a modified NGT using the RAND form (a type of the NGT developed by the RAND corporation)^35^ to facilitate discussion, the findings of Phases 1 and 2 will be reviewed and consolidated to produce recommendations on how to reach each persona.

#### Recruitment to consensus meeting

The target attendance will be an odd number of attendees between 9–16 in total. This will include the steering group who will form no more than half of the attendees. The additional attendees will be selected from the following groups: GPs/ Neurologists/ AHPs/ spinal surgeon/ educationalist (a professional who specialises in education pedagogy, or with experience in delivering educational interventions), and a patient representative. We will aim for a 50:50 gender balance. Attendees will be recruited from the RECODE-DCM community, and from previous participants involved in Phases 1 and 2.

#### Consensus meeting process

Prior to the consensus meeting, the management group will review the results of phases 1 and 2, and generate potential educational interventions which will be pre-circulated to the meeting attendees. In this survey, the participants will be asked to vote on the appropriateness (Likert scale, 1=Very inappropriate; and 5=uncertain/equivocal; 9=very appropriate) of a particular educational intervention for the described persona. The meeting will be held virtually on a videoconferencing application, and will be facilitated by an independent facilitator. The Management Group will present the reasons for including each educational intervention. The aggregate results of the pre-circulated anonymous survey will be presented to the attendees, who will then be invited to respond to the selection discrepancies. Attendees will then be asked to privately re-rank the appropriateness of the educational intervention using re-circulation of a virtual survey. If there is more than one persona, they will be asked to rank one educational intervention for each persona. IM principles will be applied to identify the determinants which may impact the effective delivery of the educational intervention to each persona.

#### Analysis

The median rating will be used as the appropriateness score. To analyse the results, we will apply a binomial distribution. The definitions for appropriateness of the intervention will be as follows: (i) appropriate=median of 7–9 without disagreement; (ii) uncertain/equivocal=median of 4–6 OR if disagreement exists; (iii) inappropriate=median of 1–3, without disagreement.^44^ If nine members attend the meeting, agreement will be defined as when no more than two individuals rate an intervention outside a specified 3-point range (1–3, 4–6, 7–9), while disagreement will be defined as when three or more individuals rate an intervention in the 7–9 range, and another three rate the same intervention in the 1–3 range.^35^ The outlined methodology introduces bias when the panel size exceeds nine members. Notably, the method proves effective for panel sizes divisible by three, as it maintains a consistent criterion for disagreement: a minimum of one-third of panel members indicating extreme ratings (1–3 OR 7–9). Nonetheless, as the number of members increases, the degree of disagreement may be amplified.^44^ An alternative methodology is proposed to address this issue using the interpercentile range adjusted for symmetry (IPRAS) for statistical evaluation of agreement or disagreement. The interpercentile range (IPR) defining disagreement is observed to be larger than the IPRAS in symmetric distributions around the median rating on a 9‐point scale (median=5, therefore a symmetrical four ratings above and below the median).^44 45^ The methodology to calculate the IPR and IPRAS is detailed in the RAND user manual.^44^

The outcome of this consensus meeting will be to use the results of Phase 2 to recommend a potential educational intervention based on the systematic approach for piloting during phase 3.

### Phase 3: evaluation and pilot implementation

Aim: To develop a pilot educational intervention using the developed framework.

Deliverable: An educational intervention piloted on a group of professionals.

To investigate the efficacy of our framework, we will co-design an educational intervention based on the results of phases 1 and 2. This intervention will be created from the systematic framework and will be used to target our identified personas within the DCM diagnostic pathway. We will compare/contrast to previous group experience,^26^ to establish if the intervention is effective. The steering group will meet in a consensus meeting, and the results from Phases 1 and 2 will be combined. We will decide on an educational intervention that targets a persona as identified in Phase 1, which is relevant to the current trends in resource availability as identified in Phase 2. The criteria for this decision will include considerations of the likely efficacy, affordability, practicality and scalability of the intervention. We will employ IM at this stage to ensure the educational content is focused, evidence-based and designed to effectively change practice behaviour. The intervention will have a follow-up phase at 6–12 months to assess for knowledge retention and changes in referral/diagnostic behaviour. This detail will be published in advance of the intervention.

## Discussion

This protocol describes a comprehensive and iterative process to establish an educational framework to accelerate diagnosis in DCM. The process will establish exactly ‘who’ requires ‘what’ information and ‘how’. It will also aim to consolidate these requirements into common groups (personas), such that the created educational resources, strategies or interventions can be reused for greater efficiency.

### Why is this foundational process required?

Currently, patients need to see multiple healthcare providers before receiving their DCM diagnosis.^20^ Given the impact of diagnostic delay on the outcome of patients with DCM, it is essential to educate healthcare providers to help them identify the relevant signs and symptoms of DCM and understand the proper diagnostic workup for this condition. However, drawing from our previous research,^26^ we understand that addressing this requires an investigation of the target population, their information needs and preferred learning methods. We include a thorough investigation of the target population through the development of educational personas. This is extremely important to elucidate prior to developing the educational intervention. This process may only reveal one or perhaps several personas. Nonetheless, it is essential to define the personas to ensure that the educational content delivery is targeted and appropriate for each professional who uses our educational intervention. Indeed, this may result in the creation of more than one educational intervention to fit the different personas. The outputs of our foundational process will provide insights into these pertinent questions, which will subsequently shape our intervention. This approach will ensure that our intervention is developed to meet the needs of our target audience. It aims to ensure efficiency and effectiveness.

### Is our methodology appropriate?

A central methodology is the Delphi method; an iterative process that starts with scoping exercises followed by gradual distillation of information using a representative panel of experts. Previous studies have shown that defining a set of core competences for education can inform national policy and guidance. The British Society of Antimicrobial Chemotherapy (BSAC) created a set of core competencies for undergraduate medical education on antimicrobial stewardship using the Delphi method,^46 47^ which were subsequently endorsed by the National Institute for Health and Care Excellence (NICE).^48 49^ This highlights the efficacy of the Delphi method in shaping educational competencies through consensus, and the national relevance and importance of its outputs.

Our methodology also uses adaptations of the NGT. Both the Delphi method and NGT are established methods of reaching group consensus^35^ that capture a range of expert opinions. While we value the broader reach of an electronic Delphi, these methods are time and resource intensive to conduct and risk fatiguing a community with multiple surveys. The use of an NGT methodology, with an expert group carefully put together in the Steering Committee, therefore represents a pragmatic step to balance these risks.

Ultimately, this is a novel approach to create an educational strategy. It aims to take a very thorough and ground-up approach, to ensure maximum efficiency and effectiveness. We have built on our experience of running such projects globally, through the AO Spine RECODE-DCM Project. Given the lack of conclusive evidence to guide educational intervention development in this domain, our consensus-based approach will incorporate opinions from a multidisciplinary cohort formed of clinicians with direct experience in managing the condition, researchers with relevant theoretical expertise, and patients with lived experience. To ensure that diverse perspectives contribute to the consensus, we will aim for gender parity and will also solicit international stakeholder participation. Further, the output of our educational framework will be piloted in phase 3, with the results informing the appropriateness of our methods in achieving our desired output. The pilot phase also provides the opportunity to further iterate our intervention before releasing it to our target population.

### What are the limitations?

We acknowledge certain limitations to our methodology, relevant to survey methods, its setting and the iterative nature of this project. With respect to the Delphi method, there may be a bias in the descriptors generated due to the composition of the panellists and the potential for high dropout rates across successive rounds of the survey. To account for this, we will be employing the modified Delphi approach, using existing information to generate the initial set of descriptors. This will reduce the bias by systematically generating the initial descriptors and will potentially also reduce the number of survey rounds needed, thus addressing the potential for a high dropout rate during successive rounds of the survey.

Relevant to the study setting, we have focused on HIC, which may limit the universal applicability of the consensus generated. In an attempt to address this, we have mandated inclusion of international participants to account for global differences in healthcare delivery. The rationale for this approach, as explained within the introduction, is to ensure consistency in access to the required surgical intervention. Future work, once the intervention has been developed and piloted, will include collaborations addressing priorities in low and middle-income countries, accounting for the differences in resource availability. Finally, we recognise that certain aspects of this study are undefined and may require updates as the study proceeds. Specifically, as we acquire the knowledge of the information needs of our population, we may need to adjust our intervention delivery approach accordingly. We have therefore outlined how each phase may be conducted; however, we recognise that some iterative changes may be necessary as the results emerge. A further limitation (as currently planned) is that knowledge/competency acquisition will be evaluated. To be effective as an intervention, clinicians must implement this in practice. It will be important to evaluate the implementation of the learning in practice, and also the impact on patient outcomes of this; however, this is outwith the scope of this project.

Long-term physical impairment from DCM is preventable in the context of early diagnosis and early access to definitive specialist management. This protocol describes a comprehensive three-phase mixed-methods approach, designed to systematically develop and pilot an educational framework aimed at producing an educational intervention for professionals involved within the DCM diagnostic pathway. In the long term, our framework to design the educational intervention will generate reusable resources and will also serve as a blueprint for further pedagogy research, relevant to other diseases that require similar educational interventions in an environment where resources are limited and efficiency in targeting a broad population is paramount.

## Supplementary material

10.1136/bmjopen-2025-107940 online supplemental file 1
