# Supplementary figures and images for: Accelerating diagnosis of degenerative cervical myelopathy through improved education: a mixed-methods study protocol from Myelopathy.org RECODE-DCM to define stakeholders, knowledge requirements and an optimal intervention strategy
Source: BMJ Open. 2026 Mar 24;16(3):e107940. doi: 10.1136/bmjopen-2025-107940 (PMC13034346; doi:10.1136/bmjopen-2025-107940)

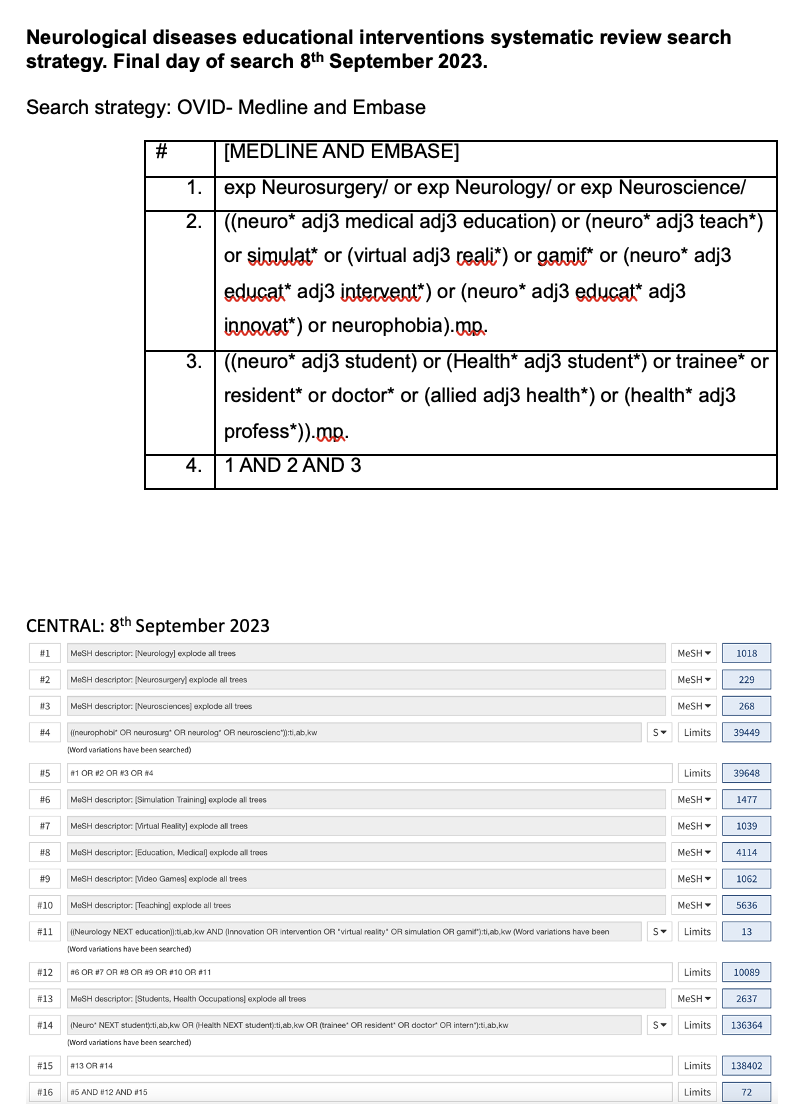
Supplementary data

**Supplementary Figure 1** Search strategy

Supplement: online supplemental file 1 [file bmjopen-16-3-s001.docx]
