# Supplementary material for: Accelerating diagnosis of degenerative cervical myelopathy through improved education: a mixed-methods study protocol from Myelopathy.org RECODE-DCM to define stakeholders, knowledge requirements and an optimal intervention strategy
Source: BMJ Open. 2026 Mar 24;16(3):e107940. doi: 10.1136/bmjopen-2025-107940 (PMC13034346; doi:10.1136/bmjopen-2025-107940)
Supplement: Supplementary file 2 [file bmjopen-2025-107940.reviewer_comments.pdf]

PEER REVIEW HISTORY

BMJ Open publishes all reviews undertaken for accepted manuscripts. Reviewers are asked to complete a checklist review form and are provided with free text boxes to elaborate on their assessment. These free text comments are reproduced below.

ARTICLE DETAILS

Title (Provisional)

Accelerating Diagnosis of Degenerative Cervical Myelopathy Through Improved Education: A mixed-methods study protocol from Myelopathy.org RECODE-DCM to define stakeholders, knowledge requirements and an optimal intervention strategy.

Authors

Veremu, Munashe; Deakin, Naomi; Chauhan, Rohil V; Lantz, Justin M.; Toumbas, Georgios; Tabrah, Julia; Kumar, Vishal; Zipser, Carl; Plener, Joshua; Ammendolia , Carlo; Anderson, David B; dos Santos Rubio, Ellianne J.; Tetreault, Lindsay; Parnaik, Rahul; Rodrigues-Pinto, Ricardo; Ong'wen, Otieno Martin; Sarewitz, Ellen; Sadler, Iwan; Roberts , Theresa; Langridge, Neil; Swait, Gabrielle; Hadfield-Law, Lisa; Draper-Rodi, Jerry; Wood, Lianne; Stacpoole, Sybil; Ganau, Mario; Baig, Shehla; Bateman, Antony; Demetriades, Andreas; Peul, Wilco; Davies, Benjamin

---

VERSION 1 - REVIEW

**Reviewer**	**1**
**Name**	**Koyanagi, I**
**Affiliation**	**Hokkaido Neurosurgical Memorial Hospital, Sapporo, Japan**
**Date**	**11-Sep-2025**
**COI**	**None**

The authors reported the project of creating educational strategy for early diagnosis of degenerative cervical myelopathy. The project involves several specialist and non-specialist groups through three phases. The methodology shown in this manuscript is logical and reasonable. I have some concerns: The term, “degenerative cervical myelopathy” actually includes several known degenerative spinal disorders such as cervical spondylosis, cervical OPLL or cervical disc hernia. There are significant variations in clinical appearance, symptom progression and medical terms to describe this condition in clinical practice. Probably phase 1 will be important process.

**Reviewer**	**2**
**Name**	**Indira, Bhagavatula**

**Affiliation**	**National Institute of Mental Health and Neuro Sciences**
**Date**	**16-Oct-2025**
**COI**	**None**

The authors need to state clearly who and what groupthey are targetting to educate and empower- since they feel that the diagnosis is delayed resulting in less than acceptable outcomes the group may include physicians family GPS physiotherapists orthopedic etc and once diagnosis is made look for confirming withimaging and surgical management as required. .

clarity is required about the groups.

**Reviewer**	**3**
**Name**	**Korkie, Elzette**
**Affiliation**	**University of Pretoria Faculty of Health Sciences, Physiotherapy**
**Date**	**05-Nov-2025**
**COI**	**None**

A very valid and much needed study. All the best

**** The reviewer provided a marked copy with additional comments. Please contact the publisher for full details. ****

**Reviewer**	**4**
**Name**	**Nouri, Aria**
**Affiliation**	**University Hospitals of Geneva, Department of Neurosurgery**
**Date**	**24-Dec-2025**
**COI**	**None**

---

no major concerns

**Reviewer**	**5**
**Name**	**Graco, Marnie**
**Affiliation**	**Institute for Breathing and Sleep, Institute for Breathing and Sleep**
**Date**	**25-Jan-2026**
**COI**	**None**

This is an important program of research, aiming to meet an unmet need in clinical care. DCM is a common condition which is poorly recognised and diagnosed. The authors have made a strong

rationale for their program of research, which is likely to have significant impact on the outcomes of people with DCM. However, I have serious concerns about whether this program of research is suitable for publication as a protocol.

This is a long and complicated protocol, which describes the methods of not one, but a series of many (at least 8) related and highly dependent studies. I question the need to publish this as a “single protocol” and highly doubt that these studies will not change significantly as the program of research progresses. As described by the authors this is an “iterative” program; there are many contingencies and dependencies that are (appropriately) built into the methods, which almost guarantees that the project will evolve and change.

Many of these studies lack important information about the participant population, recruitment strategies, data collection procedures, analysis methods, anticipated timelines, etc etc that would be expected in a protocol. For example, this protocol is aiming to describe the methods of the following studies in one paper:

- Phase 1a

- stage 1 – process mapping and patient and health professional interviews

- stage 2 – interviews and workshops with a new cohort of professionals identified in stage 1

- stage 3 – consensus meeting (nominal group technique) with the steering group

- Phase 1b – delphi study

- Phase 2a – systematic review

- Phase 2b – survey of professional development providers

- Phase 2c – consensus meeting

- Phase 3: evaluation and pilot implementation

I do not want to discourage the authors from this important work, but I cannot see the value in publishing this as a protocol.

VERSION 1 - AUTHOR RESPONSE

Reviewer: 1

Dr. I Koyanagi, Hokkaido Neurosurgical Memorial Hospital, Sapporo, Japan Comments to the Author:

The authors reported the project of creating educational strategy for early diagnosis of degenerative cervical myelopathy. The project involves several specialist and non-specialist groups through three

phases. The methodology shown in this manuscript is logical and reasonable. I have some concerns: The term, “degenerative cervical myelopathy” actually includes several known degenerative spinal disorders such as cervical spondylosis, cervical OPLL or cervical disc hernia. There are significant variations in clinical appearance, symptom progression and medical terms to describe this condition in clinical practice. Probably phase 1 will be important process.

- *We thank the reviewer for their thoughtful comments and agree that Phase 1 is a critical component of this project. Regarding the nomenclature of degenerative cervical myelopathy, the use of this unifying term is based on prior international, multi-stakeholder consensus work conducted through AO Spine RECODE-DCM (Davies BM, Khan DZ, Barzangi K, et al. We Choose to Call it 'Degenerative Cervical Myelopathy': Findings of AO Spine RECODE-DCM, an International and Multi-Stakeholder Partnership to Agree a Standard Unifying Term and Definition for a Disease. Global Spine J. 2024;14(2):503-512. doi:10.1177/21925682221111780). This initiative recommended adoption of a single overarching term encompassing the spectrum of degenerative cervical spinal cord compression disorders to improve clarity, consistency, and research efficiency. We have therefore retained this terminology in the present study to align with established consensus and to support comparability across future research.*

Reviewer: 2

Prof. Bhagavatula Indira, National Institute of Mental Health and Neuro Sciences Comments to the Author:

The authors need to state clearly who and what group they are targetting to educate and empower- since they feel that the diagnosis is delayed resulting in less than acceptable outcomes the group may include physicians family GPS physiotherapists orthopedic etc and once diagnosis is made look for confirming with imaging and surgical management as required. Clarity is required about the groups.

- *We thank the reviewer for highlighting the importance of clearly defining the target professional groups. We agree this is critical; however, the population involved in the diagnosis of degenerative cervical myelopathy is highly heterogeneous across healthcare systems. To address this, Phase 1a (Stage i) of the study includes a structured prioritisation process to identify and define the key stakeholder groups to be targeted in subsequent phases. Therefore, the specific target audience cannot be prespecified at this stage, and determining this is a primary objective of the first phase of the study.*

Reviewer: 3

Dr. Elzette Korkie, University of Pretoria Faculty of Health Sciences Comments to the Author:

A very valid and much needed study. All the best. Please see attached file for in-text comments.

- *We thank the reviewer for the comments. In text comments have been addressed.*

Reviewer: 4

Dr. Aria Nouri, University Hospitals of Geneva Comments to the Author:

no major concerns

- *We thank the reviewer for their comments.*

Reviewer: 5

Ms. Marnie Graco, Institute for Breathing and Sleep, The University of Melbourne Comments to the Author:

This is an important program of research, aiming to meet an unmet need in clinical care. DCM is a common condition which is poorly recognised and diagnosed. The authors have made a strong rationale for their program of research, which is likely to have significant impact on the outcomes of people with DCM. However, I have serious concerns about whether this program of research is suitable for publication as a protocol.

This is a long and complicated protocol, which describes the methods of not one, but a series of many (at least 8) related and highly dependent studies. I question the need to publish this as a "single protocol" and highly doubt that these studies will not change significantly as the program of research progresses. As described by the authors this is an "iterative" program; there are many contingencies and dependencies that are (appropriately) built into the methods, which almost guarantees that the project will evolve and change.

Many of these studies lack important information about the participant population, recruitment strategies, data collection procedures, analysis methods, anticipated timelines, etc etc that would be expected in a protocol. For example, this protocol is aiming to describe the methods of the following studies in one paper:

- Phase 1a
- stage 1 – process mapping and patient and health professional interviews
- stage 2 – interviews and workshops with a new cohort of professionals identified in stage 1 - stage 3 – consensus meeting (nominal group technique) with the steering group
- Phase 1b – delphi study
- Phase 2a – systematic review
- Phase 2b – survey of professional development providers - Phase 2c – consensus meeting
- Phase 3: evaluation and pilot implementation

I do not want to discourage the authors from this important work, but I cannot see the value in publishing this as a protocol.

- *We thank the reviewer for their thoughtful and constructive comments and for recognising the importance of this research programme. We acknowledge that this is a complex, multi-phase and iterative protocol. This design is intentional, as the framework aims to address an area without clear methodological precedent and therefore requires flexibility to allow findings from earlier phases to inform subsequent study components.*
- *For this reason, we have presented a unified protocol describing the overall framework and methodological principles, rather than multiple highly prescriptive individual protocols that may become inaccurate as the programme evolves. Where detailed methodological elements depend on outputs from earlier phases (for example, identification of target professional groups in Phase 1 informing recruitment in later phases), these cannot be fully specified a-priori; instead, we provide transparent processes for how such decisions will be made.*
- *In response to the reviewer's concerns, we have clarified methodological descriptions where possible by added definitions to for the terms 'Educational strategy', 'Educational framework' , and we have added anticipated project timelines to improve transparency. We believe publication of this overarching protocol provides value by defining a novel, reproducible framework for developing educational interventions in DCM and other conditions requiring educational interventions.*
